# Supplementary material for: 1,2,3,4,6-O-Pentagalloylglucose Protects against Acute Lung Injury by Activating the AMPK/PI3K/Akt/Nrf2 Pathway
Source: Int J Mol Sci. 2022 Nov 20;23(22):14423. doi: 10.3390/ijms232214423 (PMC9699101; doi:10.3390/ijms232214423)
Supplement: Supplementary file 1 [file ijms-23-14423-s001.zip › ijms-1881552-supplementary.pdf]

1,2,3,4,6-O-Pentagalloylglucose Protects Against Acute Lung Injury by Activating the AMPK/PI3K/AKT/Nrf2 Pathway

1. Isolation and identification of PGG

As the compound 1 was the constituents of *Paeonia suffruticosa* Andr. (Paeoniaceae) by HPLC, they can be separated from *Paeonia suffruticosa* Andr. (Paeoniaceae). The *Paeonia suffruticosa* Andr. (Paeoniaceae) (5 kg) were crushed and extracted with 50% EtOH for 2 h (3times). The extract was partitioned with petroleum ether, EtOAC, and n-BuOH. The n-BuOH fraction (220g) was chromatographed with an HP-20 column to give six fractions: A (H_2O), B (10% EtOH), C (30% EtOH), D (50% EtOH), E (70% EtOH) and F (95% EtOH). Fraction D was chromatographed on an RP-18 column using 0-100% MeOH to afford subfractions D1-10 via HPLC-DAD analyses. Fraction D5 (6.1 g) was chromatographed on an LH-20 column using 80% MeOH-H_2O to obtain 100 subfractions (Fr. D5.1-Fr. D5.100). Subtraction Subfraction Fr. D5.20 was purified by preparative HPLC using 30% MeOH-H_2O to yield compound 1 (600 mg). The purity analysis of PGG was by HPLC (Figure S1).

The structure of compound 1 was determined by NMR and MS spectral data. The molecular formula $C_{41}H_{32}O_{26}$ of compound 1 {m/z 939.1109 $[M-H]^-$} was determined by ESIMS and NMR data. The ^1H and ^{13}C NMR assignments of compound 1 were determined using on the basis of the 1H, 13C spectra. The ^1H and ^{13}C NMR, and ESI-MS data of 1 were identical to those of 1, 2, 3, 4, 6- pentagalloylglucose ($C_{41}H_{32}O_{26}$) in the literature (Zhang et al., 2019). ^1H and ^{13}C NMR spectral data are as follows: ^1H NMR (MEOD, 600 MHz): d 4.42 (1H, dd, J = 4.5, 10.0 Hz, glc. H-6a), 4.77 (1H, m, glc. H-5), 4.42 (1H, d, J = 10.0 Hz, glc. H-6b), 5.57 (1H, t, J = 9.0, glc. H-2), 5.61 (1H, t, J = 9.0, glc. H-4), 5.9 (1H, t, J = 9.0, glc. H-3), 6.24 (1H, d, J = 9.0, glc. H-1), 6.90, 6.95, 6.98, 7.05, 7.11 (each 2H, s, galloyl H); 13C NMR (MeOD, 100 MHz): δ (ppm):93.81 (glc. C-1), 69.79 (glc. C-4), 72.19 (glc. C-2), 74.10 (glc. C-3), 74.41 (glc. C-5), 63.11 (glc. C-6), 110.61, 110.46, 110.40, 110.37, 110.33 (galloyl C-2, C-6), 121.03, 120.34, 120.22, 120.19, 119.71 (galloyl C-1), 140.76, 140.35, 140.30, 140.12, 140.00 (galloyl C-4), 146.54, 146.46, 146.42, 146.36, 146.27 (galloyl C-3, C-5), 167.93, 167.29, 167.02, 166.92, 166.22 ($-COO^-$). The structure of compound 1 is shown in Figure S2.

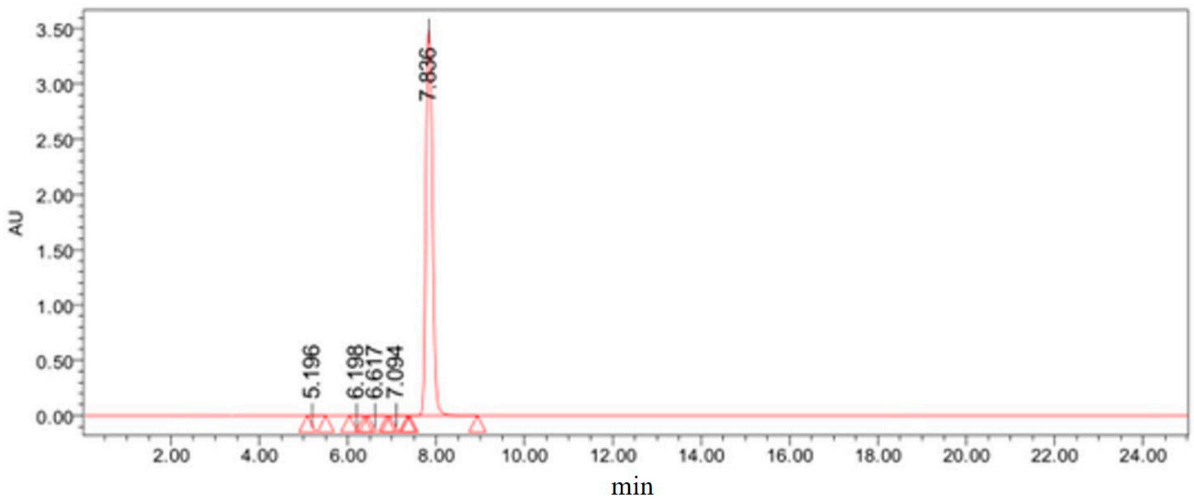

No.	Rt(min)	A%
1	5.196	0.01
2	6.198	0.01
3	6.617	0.02
4	7.094	0.01
5	7.836	99.94

Figure S1. The HPLC chromatogram of PGG

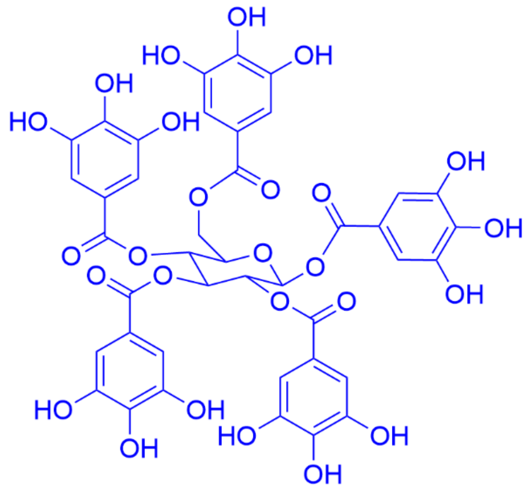

Figure S2. The structure of PGG

References

1. Zhang, Q.; Nie, J.; Chen, S.J.; Li, Q. Protective effects of ethyl gallate and pentagalloylglucose, the active components of Qingwen Baidu Decoction, against lipopolysaccharide-induced acute lung injury in rats. *Drug Des Devel Ther* **2019**, 13, 71-77, doi:10.2147/dddt.S186029.
